# Effect of Nanodiamond Incorporation on the Surface, Mechanical, and Tribological Properties of Glass Ionomer Cement

**DOI:** 10.3390/jfb17080382

**Published:** 2026-08-03

**Authors:** Magdalena Mrózek, Pavel Kejzlar, Petr Louda, Danuta Lietz-Kijak, Piotr Skomro, Karolina Jezierska, Karolina Rowińska, Lidia Szczucka, Kinga Adach, Mateusz Fijałkowski, Totka Bakalova, Helena Gronwald

**Affiliations:** 1 Department of Materials Science, Faculty of Mechanical Engineering, Technical University of Liberec, Studentská 2, 461 17 Liberec, Czech Republic; magdalena.mrozek@tul.cz (M.M.); totka.bakalova@tul.cz (T.B.); 2 Department of Advanced Materials, Institute of Nanomaterials, Advanced Technologies, and Innovation, Technical University of Liberec, Studentská 1402/2, 461 17 Liberec, Czech Republic; pavel.kejzlar@tul.cz (P.K.); kinga.adach@tul.cz (K.A.); mateusz.fijalkowski@tul.cz (M.F.); 3 Department of Material Science, Polytechnic Faculty, University of Kalisz, 2 W. Bogusławskiego Square, 62-800 Kalisz, Poland; petrlbc1@seznam.cz; 4 Department of Propaedeutic, Physical Diagnostics and Dental Physiotherapy, Pomeranian Medical University in Szczecin, 72 Powstańców Wielkopolskich Ave., 70-111 Szczecin, Poland; danuta.lietz.kijak@pum.edu.pl (D.L.-K.); piotr.skomro@pum.edu.pl (P.S.); lidia.szczucka@pum.edu.pl (L.S.); 5 Department of Medical Physics, Pomeranian Medical University in Szczecin, 13 Ku Słońcu Street, 71-073 Szczecin, Poland; karolina.jezierska@pum.edu.pl; 6 Department of Genetics and Pathomorphology, Pomeranian Medical University in Szczecin, 1 Unii Lubelskiej St., 71-252 Szczecin, Poland; karolina.rowinska@pum.edu.pl

**Keywords:** dental glass ionomer cement, nanodiamond, tribology, microhardness, wear resistance, surface roughness

## Abstract

Background/Objectives: Glass ionomer cements (GICs), high-biocompatibility dental materials that release fluoride with a cariostatic and remineralising effect on hard dental tissues, are widely used in many fields of dentistry, including conservative dentistry, pediatric dentistry, prosthodontics, and orthodontics; however, their mechanical performance and wear resistance remain limited. This study investigated the effect of nanodiamond (ND) addition (0.5–4.0 wt.%) on the surface, mechanical, and tribological properties of a conventional self-curing GIC. Methods: Surface roughness, microhardness, wear resistance, coefficient of friction, and chemical composition were evaluated using confocal microscopy, Vickers microhardness testing, tribological measurements, and EDX analysis. Results: The incorporation of NDs reduced the coefficient of friction and modified the surface morphology of the cement. The lowest friction coefficient (0.444) was observed for the sample containing 2 wt.% ND. However, ND addition also resulted in an approximately 50 per cent decrease in microhardness and wear resistance compared with the unmodified cement. EDX analysis confirmed that the chemical composition of the GIC matrix remained largely unchanged. Conclusions: The results indicate that nanodiamonds can improve the tribological behavior of glass ionomer cements, although further optimization of nanoparticle concentration and dispersion is necessary to maintain adequate mechanical properties.

## 1. Introduction

Glass ionomer cements (GIC) represent one of the most significant innovations in modern conservative dentistry. Their history dates back to 1969, when they were first developed, and their clinical application was subsequently introduced and systematically described by Wilson and Kent [1,2] in the early 1970s. Since then, GICs have been more than just a tooth restoration material characterized by high fluoride release and a white color. Its applications in dentistry include the restoration of primary teeth, class III and V of Black restorations, luting cements for fixation of prosthetic restorations, and orthodontic appliances, the restoration of non-carious lesions, temporary fillings in endodontics procedures, liner and base of restorations, and dental sealants [3,4,5].

The main advantage of glass ionomer cements is their unique combination of biological and functional properties. Their good biocompatibility makes them well tolerated by surrounding hard and soft tissues [6]. At the same time, the continuous release of fluoride ions provides long-term protection against demineralisation and acts as an effective anticariogenic agent [7]. Furthermore, although GIC cements are inherently brittle materials with lower compressive and flexural strength compared with dentine and resin composites, their coefficient of thermal expansion and modulus of elasticity are similar to those of dentine, which helps to maintain marginal integrity under thermal fluctuations and evenly distribute occlusal stresses, reducing the likelihood of crack formation within the restoration [8]. Another significant benefit is their ability to bond chemically to enamel and dentin, forming a durable chemical bond and eliminating the need for a complex, multi-step resin-based adhesive system. However, to maximise this intrinsic adhesion, manufacturers often recommend the use of a polyacrylic acid-based cavity conditioning agent (primer) to remove the smear layer and increase the surface energy of dental tissues [9]. On the other hand, glass ionomer cements suffer from low mechanical strength, inferior wear resistance, limited fracture toughness, lower aesthetic quality, and a tendency to absorb water, which can lead to their dissolution or wear [10,11,12]. In addition, their susceptibility to moisture imbalance during the early stages of setting may negatively affect their long-term stability and durability.

In conservative dentistry, GIC is the preferred material for treating caries in primary teeth and for small cavities in permanent anterior teeth, where chewing forces are not high and, consequently, the need for high mechanical strength is less critical [3]. In recent decades, various strategies have been intensively researched to increase the strength and durability of GICs. Researchers have focused primarily on modifying the chemical composition and structure of both the glass ionomer and polyalkenoic acid, as well as on the incorporation of advanced reinforcing fillers, with nanomaterials playing a significant role [2,13,14,15]. Nanotechnology has made it possible to reduce particles to sizes of 1–100 nm, which fundamentally alters their physicochemical, surface, and mechanical properties [16]. Incorporating nanoparticles into the glass phase of GICs has resulted in a broader particle size distribution and improved particle packing density, leading to higher compactness and better utilization of the cement matrix [17]. Several previous studies have demonstrated that adding various types of nanoparticles—such as TiO_2_, ZrO_2_, ZnO, or hydroxyapatite—can significantly enhance the mechanical performance of GICs. Reported improvements include increased compressive and flexural strength, greater microhardness, and reduced wear, as well as enhanced resistance to crack initiation and propagation. These improvements have been attributed to better filler–matrix interaction, decreased porosity, and the reinforcing effect uniformly dispersed nanoparticles within the cement matrix [18,19,20,21,22,23]. Moreover, nanoparticles can influence the setting reaction and microstructure development of GICs, further contributing to improved mechanical performance and long-term stability. These findings support the potential of nanotechnology to overcome some of the inherent mechanical limitations of conventional GIC formulations and to expand their applicability in clinical dentistry and restorative applications requiring improved durability and wear resistance. In this context, nanodiamonds (ND)—an allathrope of carbon and of the most promising representatives of nanocarbon materials—have attracted particular attention. Nanodiamonds are characterized by exceptional hardness, chemical stability, a high specific surface area, and unique optical properties, while their surface chemistry is highly variable and allows tailored functionalization for a wide range of biomedical and technological applications [24,25,26,27,28,29]. Furthermore, their nanoscale dimensions and large surface-to-volume ratio promote effective interaction with surrounding matrix materials, making them attractive reinforcing agents in composite systems. These characteristics make nanodiamonds an ideal nano-reinforcement for glass ionomer cements.

Therefore, this work aims to evaluate the effect of adding nanodiamonds in various weight concentrations (0.5, 1, 2 and 4 wt.%) on the key mechanical and tribological parameters of GIC. As part of the experimental programme, surface roughness, microhardness and wear resistance were determined and compared. It is hypothesized that while an appropriate concentration of nanodiamonds can effectively reduce the friction coefficient at the contact interface, this physical modification may concurrently disrupt the structural integrity of the cement matrix, leading to a measurable trade-off in microhardness and wear resistance. Since ND powder was used, an increase in load-bearing capacity, a reduction in porosity, and improved resistance to surface damage and wear are expected, which should lead to a significant improvement in the mechanical and tribological properties of the glass-ionomer cement.

The novelty of this work lies in the investigation of nanodiamonds as reinforcing nanofillers for glass ionomer cements and in the evaluation of their effect on surface roughness, microhardness, and wear resistance. The study provides new insights into the potential of nanodiamonds to enhance the mechanical and tribological performance of dental restorative materials. Recent studies have begun exploring nanodiamonds as additives in glass ionomer cements, focusing primarily on their biological and chemical interactions. For instance, Mulder et al. demonstrated that the addition of nanodiamonds significantly enhances the ion-release capabilities of GICs without compromising their antimicrobial properties against Streptococcus mutans [30]. However, the mechanical performance of these modified cements—particularly their contact mechanics—remains largely unexplored. Therefore, the novelty of this paper lies in its comprehensive evaluation of the tribological behaviour (coefficient of friction) of nanodiamond-modified GICs. Unlike previous works, this study uniquely identifies a physical trade-off between improved frictional properties and reduced microhardness and wear resistance, highlighting the critical importance of an optimal nanoparticle concentration for structural integrity.

## 2. Materials and Methods

### 2.1. Glass Ionomer Cement (GIC)

The experimental samples were prepared using a commercially available self-curing glass ionomer cement (GIC; SDI Riva Self Cure, SDI Limited, Bayswater, VIC, Australia), which is supplied as a two-component system consisting of a powder and a liquid phase. The powder component primarily contains silicon oxide, calcium chloride, sodium fluosilicate, and aluminium phosphate. The liquid component is composed of polyacrylic acid, tartaric acid, and deionised water. These constituents undergo an acid–base setting reaction, resulting in the formation of a hardened cement matrix with adhesive and bioactive properties.

### 2.2. Modification of Glass Ionomer Cement

Glass ionomer cement (GIC) was experimentally modified by incorporating detonation nanodiamond (ND) powder (Adamas Nanotechnologies, Raleigh, NC, USA; size 30–35 nm) into the powder component of the cement. The ND powder was homogenised with the GIC powder using a dual-stage protocol to maximize dispersion: initial manual mixing in an agate mortar and pestle for 5 min, followed by 5 min of mechanical dispersion using an ultrasonic powder mixer (total mixing time: 10 min). To minimise moisture absorption by the hygroscopic GIC powder during sample preparation, all mixing procedures were carried out in a laboratory fume cupboard under controlled laboratory conditions. The modification was performed by adding nanodiamonds at different weight concentrations (wt.%) relative to the total mass of the powder component. After thorough homogenization, the modified powder mixtures were stored in their original airtight containers together with silica gel desiccant to minimise moisture uptake and prevent premature degradation of the material. All containers were hermetically sealed and stored under laboratory conditions until sample preparation.

The experimental design consisted of five groups:

Group 1 (Control): Unmodified glass ionomer cement (reference material);
Group 2: GIC containing 0.5 wt.% nanodiamond powder;
Group 3: GIC containing 1.0 wt.% nanodiamond powder;
Group 4: GIC containing 2.0 wt.% nanodiamond powder;
Group 5: GIC containing 4.0 wt.% nanodiamond powder.

While the control group (Group 1) maintained the manufacturer’s recommended 1 scoop to 1 drop dispensing ratio (corresponding exactly to 0.22 g of GIC powder mixed with 0.06 g of liquid), the modified mixtures contained the same baseline masses plus the supplementary nanodiamond mass. Specifically, the exact mass of nanodiamonds added to 0.22 g of GIC powder was: 0.0011 g for Group 2 (0.5 wt.%), 0.0022 g for Group 3 (1.0 wt.%), 0.0044 g for Group 4 (2.0 wt.%), and 0.0088 g for Group 5 (4.0 wt.%). All modified powder mixtures were subsequently mixed with a constant 0.06 g of liquid, thus progressively increasing the true powder-to-liquid mass ratio and reducing the relative volume of liquid available for the setting reaction.

The selected nanodiamond concentrations were chosen to evaluate the influence of nanoparticle content on the mechanical and tribological behaviour of the glass ionomer cement and to identify the concentration providing the most favourable balance of properties.

### 2.3. Preparation of Samples

Using a plastic spatula, the mixture was prepared by manually mixing the powder and liquid components at a powder-to-liquid weight ratio of 1:1 on a glass plate. The mixing procedure was performed by a single trained operator under standard laboratory conditions (23 ± 1 °C) and in accordance with the manufacturer’s instructions. Immediately after preparation, the homogeneous cement paste was transferred into two types of silicone moulds: (i) disc-shaped moulds (10 mm in diameter and 2 mm in height); and (ii) cuboid-shaped moulds (20 mm× 10 mm × 4 mm; length × width × height). Each mould was placed between two smooth glass plates. Before contact with the cement, the surface of the glass plates was wiped with a gauze pad soaked in a single dose of KaVo Spray (KaVo Dental GmbH, Biberach, Germany), to act as a barrier. This prevented adhesion and ensured an intact, smooth sample surface.

A total of 45 samples (9 per experimental group) were prepared. Within each group, 6 disc-shaped specimens were utilized sequentially for non-destructive surface roughness evaluation followed by microhardness testing. The remaining 3 cuboid-shaped specimens per group were dedicated exclusively to the tribological sliding wear tests. EDX mapping was performed representatively on the reverse side of one selected cuboid specimen per group after it had undergone tribological testing.

The sample was then subjected to a constant mechanical load of 0.5 kg (yielding approximately 5 N of force) for 6 min to ensure uniform sample geometry and complete material setting. After curing, the samples were carefully removed from the moulds using tweezers, and excess material extending beyond the mould edges was carefully trimmed using a scalpel (Swann-Morton, Sheffield, UK). No additional surface treatment, such as grinding or polishing, was performed in order to preserve the original surface morphology of the samples. The prepared specimens were subsequently stored in Eppendorf microcentrifuge tubes containing 100 µL of distilled water to maintain a high-humidity environment and prevent dehydration during maturation. A total of 45 samples (*n* = 45) were stored at room temperature (RT) for 7 days prior to further characterization and testing. This storage period was selected to allow complete maturation of the glass ionomer cement and stabilization of its mechanical properties before evaluation.

### 2.4. Chemical Properties

The chemical composition of the tested samples was evaluated using energy dispersive X-ray spectroscopy (EDX) integrated with a Carl Zeiss ULTRA Plus scanning electron microscope (SEM) (Carl Zeiss Microscopy GmbH, Jena, Germany). The analysis was focused on determining the elemental composition of individual sample groups and assessing the presence and relative abundance of both major and minor elements. Particular attention was paid to identifying potential changes in elemental distribution associated with the incorporation of nanodiamond particles into the glass ionomer cement matrix. EDX measurements were performed under standard operating conditions using an appropriate accelerating voltage, beam current, and working distance to optimize the excitation and detection of characteristic X-rays emitted by the analyzed elements. An Ultim Max 100 energy-dispersive X-ray spectroscopy (EDS) detector was used for elemental analysis. The acquired spectra were subsequently processed using the AZtec 6.2 software package (Oxford Instruments, UK), and the elemental composition was expressed in terms of weight percentage (wt.%) and atomic percentage (at.%), where applicable For the EDX analysis, *n* = 1 representative sample from each group was evaluated.

### 2.5. Surface Roughness

Surface roughness was analysed using a SENSOFAR S neox confocal microscope (Sensofar HQ company, Terrassa, Spain) at 20× magnification. The acquired surface topography data were processed using instrument’s integrated analysis software, which was used to determine the areal roughness parameter Sa in accordance with ISO 25178-2:2021 [31], the international standard for three-dimensional surface texture characterization. To facilitate comparison with previously published studies, the profile roughness parameter Ra was also evaluated using Gwyddion software version 2.65 (Czech Metrology Institute, Brno, Czech Republic) [32], an open-source platform for visualization and quantitative analysis of surface topography data. The conversion from areal data to profile data enabled direct comparison with studies reporting conventional two-dimensional roughness parameters. Measurements were performed on six specimens from each experimental group, with two independent scans acquired per specimen—specifically, one on the top surface and one on the bottom surface. Due to the non-homogeneous nature of the material, the opposite sides were treated as independent topographical data points. All measurements were carried out on the same surface subsequently used for microhardness and tribological testing, ensuring a direct correlation between surface topography and the evaluated mechanical and tribological properties.

### 2.6. Microhardness

For microhardness evaluation, six disc-shaped specimens (10 mm in diameter and 2 mm in thickness) were prepared for each experimental group. After removal from the moulds, the samples were stored for 7 days in a high-humidity environment to allow complete maturation of the glass ionomer cement prior to testing. No additional surface treatment, such as grinding or polishing, was performed before the measurements in order to preserve the original surface characteristics of the material. Microhardness measurements were carried out using a Duramin-40 microhardness tester (Struers, Ballerup, Denmark). The Vickers indentation method was selected to evaluate the surface mechanical properties of the prepared specimens. The tests were performed using a load of 100 g (HV 0.1) with a dwell time of 10 s. A total of 12 indentations were performed for each experimental group, distributed over the sample surface to ensure representative results. The dimensions of the indentation diagonals were recorded digitally and initially evaluated using the instrument’s integrated software. Where necessary, the automatically detected diagonal lengths were verified and corrected manually to improve measurement accuracy. The Vickers microhardness values (HV) were subsequently calculated automatically by the software DuraSoft (Struers, Ballerup, Denmark) based on the measured diagonal lengths. The final microhardness values were expressed as mean values accompanied by the corresponding standard deviations.

### 2.7. Tribology Testing

The tribological properties of the prepared specimens were evaluated using a TRB3 tribometer (Anton Paar, Graz, Austria). The instrument enables tribological testing under both dry and lubricated conditions and is designed in accordance with ASTM G99-17 [33] and ASTM G133-05(2016) standards [34]. Tribological experiments can be performed using either the Ball-on-Disc configuration or a linear reciprocating sliding mode. In the present study, the linear reciprocating tribological test configuration was selected. The cuboid-shaped specimens (20 mm × 10 mm × 4 mm; length × width × height) were mounted in a dedicated sample holder and secured using two mechanical clamps to ensure positional stability throughout the test. The counterbody consisted of a 6 mm diameter aluminium oxide (Al_2_O_3_) ceramic ball, which was rigidly fixed to prevent unwanted rotation during the experiment and to ensure reproducible contact conditions. This specific reciprocating sliding wear setup and parameter selection have been previously validated and extensively described in the literature concerning the tribological evaluation of modified glass-ionomer cements [35,36,37]. For the tribological tests, *n* = 3 cuboid-shaped samples from each group were used, and tribological tests were performed under a normal load of 10 N, with a sliding speed of 47 mm/s, a stroke length of 18 mm, a total of 832 cycles, yielding a total sliding distance of 30 m. The total test duration was corrected to 1005 s per sample. The steady-state coefficient of friction (CoF) was calculated by averaging the data from the final 800 s/80% of the test duration.

All measurements were conducted under laboratory conditions at ambient temperature. The coefficient of friction (CoF) was continuously recorded throughout the experiment and automatically processed using the instrument’s dedicated software. Following the tribological tests, the wear tracks were characterized using a SENSOFAR S neox confocal microscope. The analysis focused on the determination of wear track width and maximum wear track depth, which were used as indicators of the material’s wear resistance. These parameters enabled quantitative comparison of the tribological performance of the individual experimental groups.

### 2.8. Statistical Analysis

For microhardness evaluation, individual indentation values were averaged to yield a single representative mean value per specimen prior to statistical testing to avoid pseudo-replication. Conversely, surface roughness scans from the distinct top and bottom faces of the specimens were treated as independent observations. Data tabulation and initial consistency checks were performed using Microsoft Excel 365 (Microsoft Corp., Redmond, WA, USA). All subsequent statistical evaluations, including the one-way analysis of variance (ANOVA) and Tukey’s Honestly Significant Difference (HSD) post hoc tests, were executed using MATLAB software version R2024b (MathWorks, Natick, MA, USA). Normality of the data was assessed using the Shapiro–Wilk test. The results are presented as mean values ± standard deviations (SD). A significance level of *p* < 0.05 was considered to indicate statistical significance for all evaluated parameters. Statistical significance results are indicated using the following symbols:

- ns—non-significant differences
*—*p* < 0.05 (statistically significant)
**—*p* < 0.01 (high statistical significance)
***—*p* < 0.001 (very high statistical significance)

## 3. Results

### 3.1. Elemental Analysis

**Figure 1:**
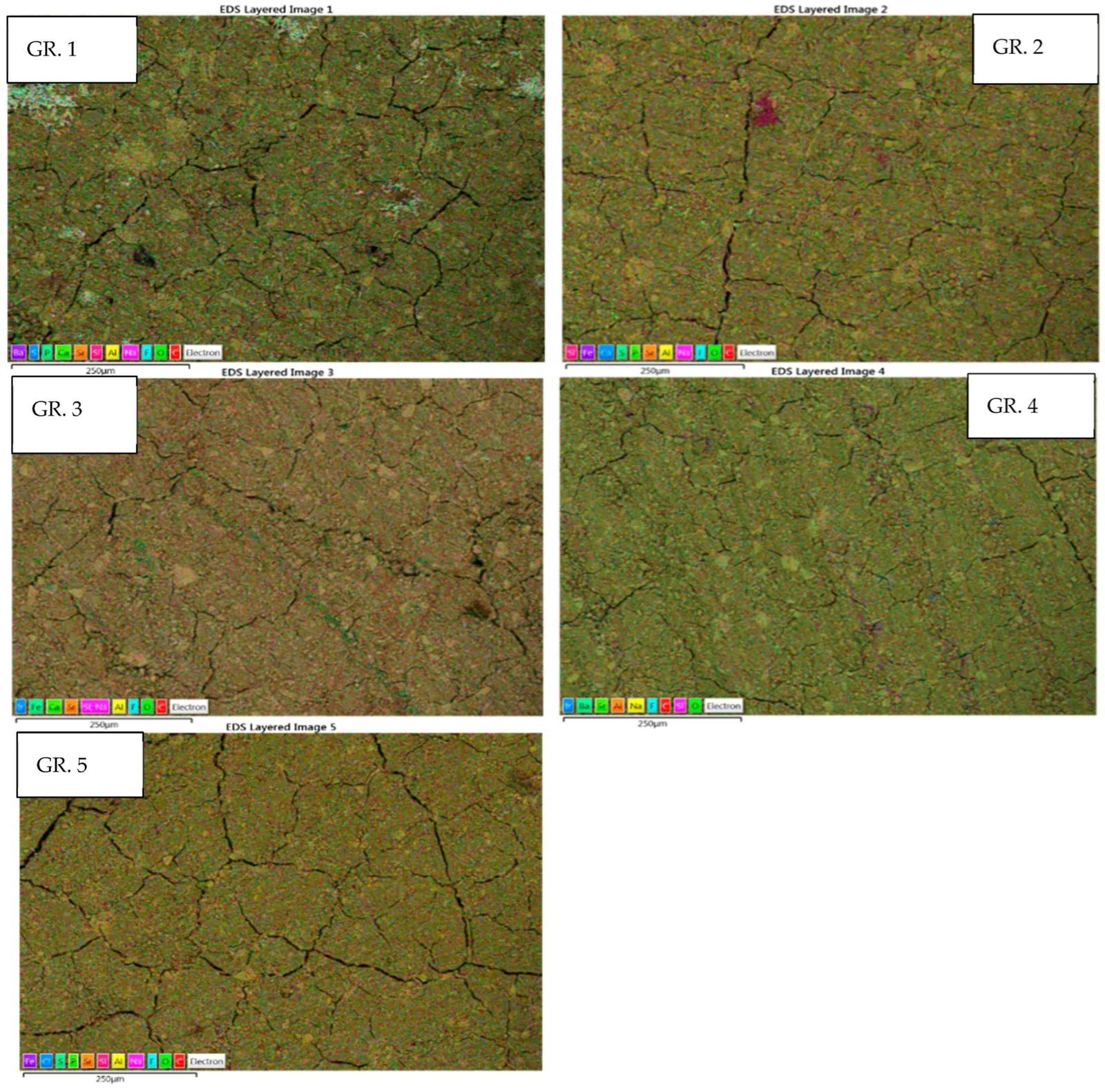
The content of individual elements determined by EDX analysis in glass ionomer cements modified with various concentrations of nanodiamonds.

Elemental analysis performed using energy dispersive X-ray spectroscopy (EDX) revealed that the primary components of all tested samples were carbon (C), oxygen (O), aluminium (Al), silicon (Si), and strontium (Sr). The relative proportions of these elements were comparable across all experimental groups, indicating that the incorporation of nanodiamonds did not significantly alter the overall chemical composition of the glass ionomer cement matrix (Figure 1). Fluorine (F), which contributes significantly to the bioactivity and anti-cavity properties of GIC, remained relatively stable in all samples, ranging from 7.24 to 8.84% wt.%. While this single-sample EDX mapping provides illustrative evidence that the principal elemental distribution remains stable, it cannot definitively confirm unchanged bulk matrix chemistry, serving rather as a preliminary indicator.

In addition, trace amounts of chromium (Cr), iron (Fe), and chlorine (Cl) were detected; however, their concentrations were very low, remaining below 0.1 wt.% in all groups. These elements are likely associated with impurities originating from raw materials or sample preparation and are not expected to influence the overall properties of the investigated cements (Table 1).

### 3.2. Vickers Microhardness

**Figure 2:**
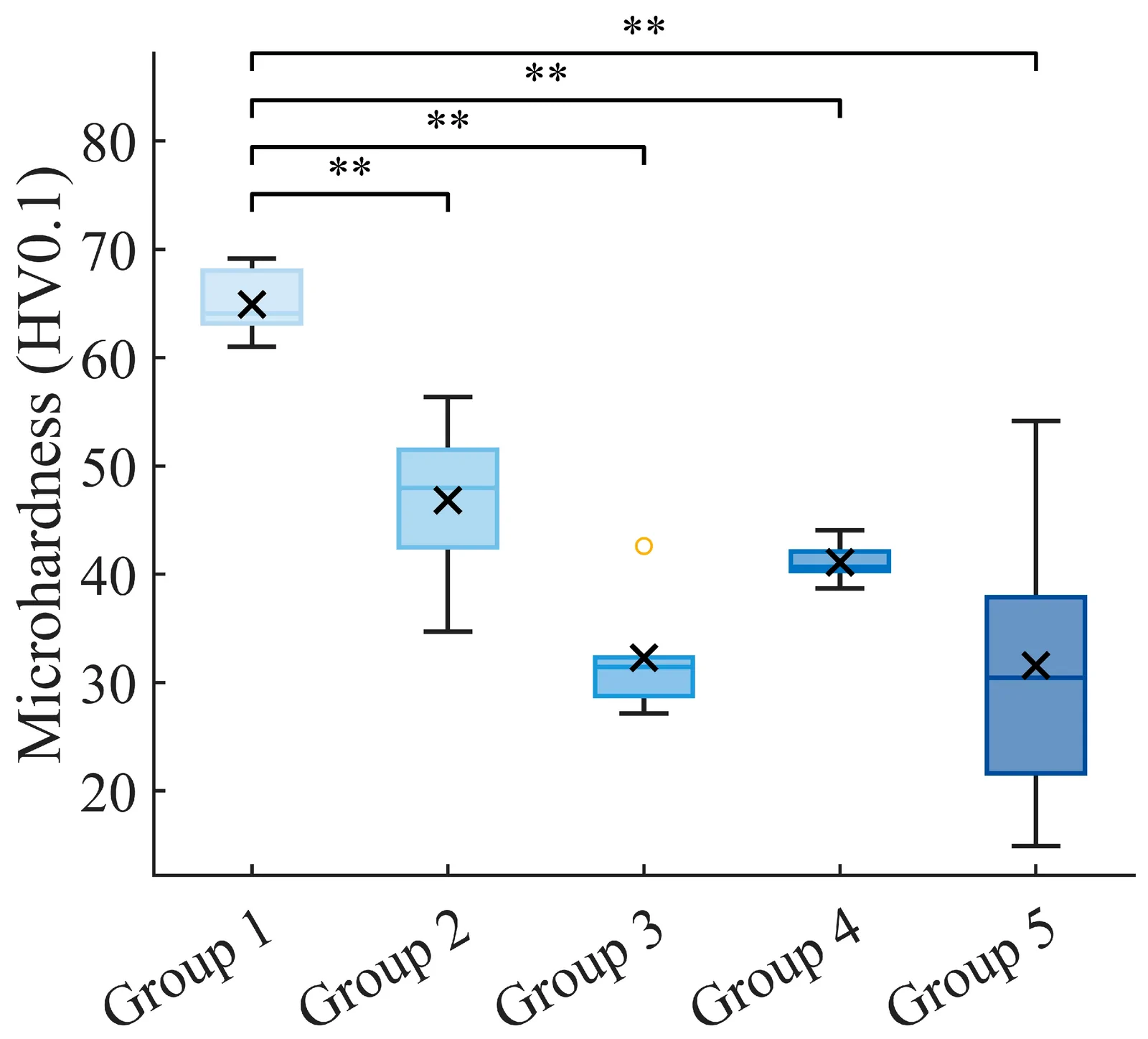
Graphical illustration of the microhardness of samples. **—*p* < 0.01 (high statistical significance); °—indicates outliers; ×—indicates the mean.

Vickers microhardness measurements (HV0.1) (Figure 2) demonstrated statistically significant differences among the investigated groups, as confirmed by one-way ANOVA model (*F*(4,25) = 18.96, *p* = 2.795 × 10^−7^, *η*^2^ = 0.75) indicating a large effect size. In particular, the unmodified glass ionomer cement (group 1) exhibited significantly higher microhardness values than all nanodiamond-modified formulations (groups 2–5; *p* < 0.01). Post hoc Tukey’s HSD analysis confirmed that microhardness of each modified group was significantly lower than that of the reference material (*p* < 0.05). The highest average microhardness was recorded for the control group (group 1), reaching 64.91 ± 3.10 HV, whereas the most pronounced reduction was observed in group 3 (1 wt.% ND) and group 5 (4 wt.% ND), with average values of 32.29 ± 5.42 HV and 31.58 ± 13.97 HV respectively (Table 2). Compared with the control material, these values represent a decrease in microhardness of approximately 50%, indicating that nanodiamond incorporation did not contribute to surface hardening under the investigated conditions. Furthermore, groups 2 and 5 exhibited the largest standard deviations, indicating greater variability in the measured values. This increased scatter may be associated with local heterogeneities in nanoparticle distribution, variations in the setting process, or differences in the microstructure of the modified cement matrix.

### 3.3. Surface Topography

Surface topography was characterised using both the areal roughness parameter (Sa) and the profile roughness parameter (Ra).

**Figure 3:**
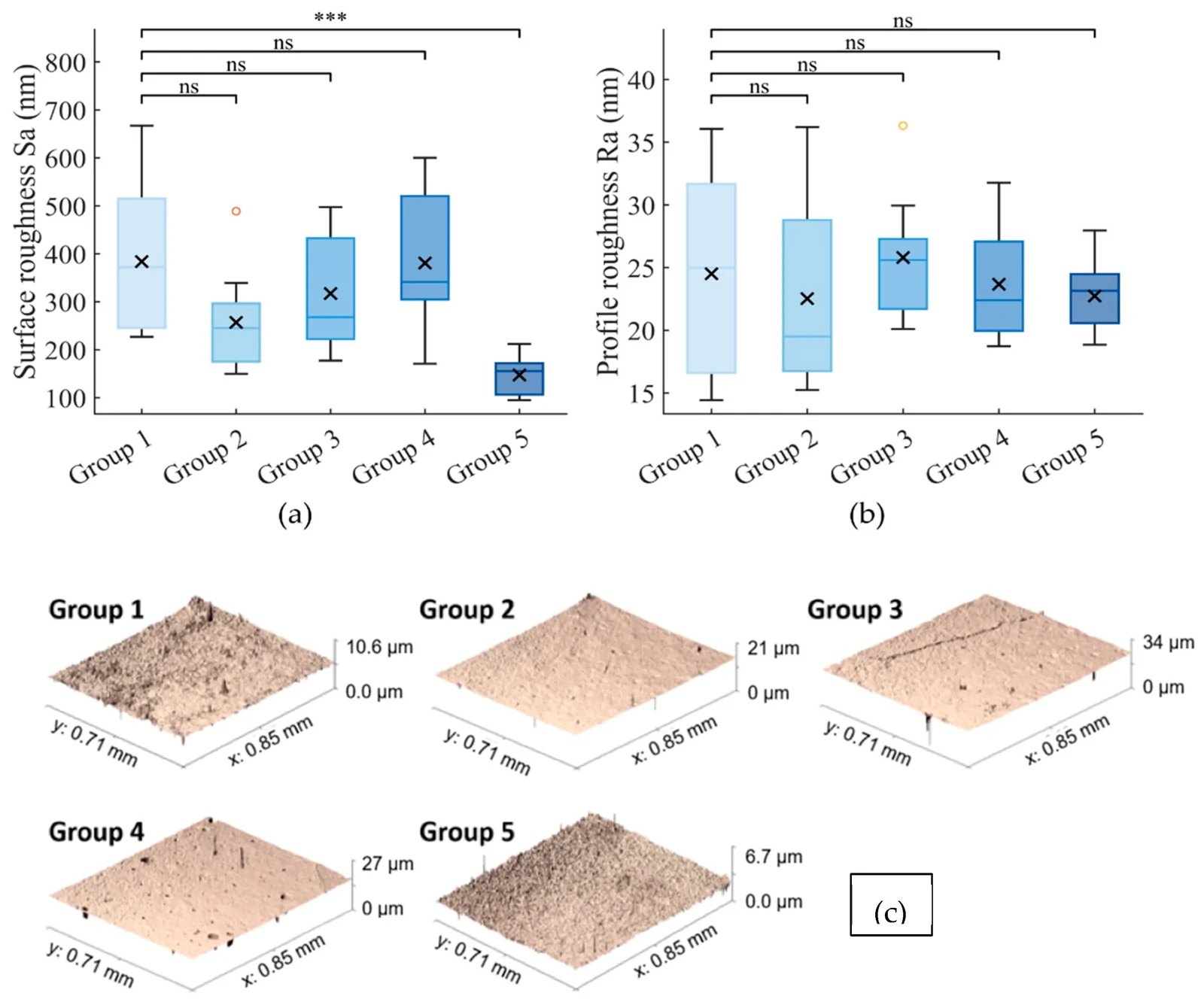
Graphical illustration of (**a**) Sa of samples; (**b**) Ra of samples; (**c**) 3D-topographical images enhancing the visual representation of the morphological changes. ***—*p* < 0.001 (high statistical significance); ns—non-significant differences; °—indicates outliers; ×—indicates the mean.

Sa (Figure 3a): One-way ANOVA revealed a statistically significant effect of nanodiamond incorporation on the areal surface roughness Sa (*F*(4,45) = 6.79, *p* = 0.000231, *η*^2^ = 0.38), indicating a large effect size. Subsequent Tukey’s HSD post hoc analysis demonstrated that a statistically significant difference was observed only between the control group (group 1) and group 5 (4 wt.% ND; *p* = 0.00058), whereas no significant differences were found between group 1 and groups 2–4 (*p* > 0.05). The highest mean Sa value was recorded for group 1 (383.7 ± 154.3 nm), followed closely by group 4 (380.8 ± 142.1 nm). Intermediate values were observed for groups 2 and 3, reaching 257.1 ± 102.9 and 317.3 ± 123.7 nm, respectively. The lowest areal roughness was measured for group 5 (147.5 ± 42.5 nm). The substantial reduction in Sa observed at the highest nanodiamond concentration suggests that nanodiamond particles may contribute to a more compact surface structure and a partial reduction in surface irregularities. In addition, groups 1, 3, and 4 exhibited relatively large standard deviations, indicating greater variability in surface morphology within these groups.

Ra (Figure 3b): In contrast to the Sa results, no statistically significant differences were detected in the profile roughness parameter Ra among the investigated groups, as confirmed by one-way ANOVA (*F*(4,45) = 0.53, *p* = 0.7165, *η*^2^ = 0.045), indicating a small effect size. Likewise, all pairwise comparisons performed using Tukey’s HSD test were statistically non-significant (*p* > 0.05). The highest mean Ra value was recorded for group 3 (25.8 ± 4.7 nm), while the lowest value was observed for group 2 (22.5 ± 7.5 nm). The relatively narrow range of Ra values and the absence of significant differences indicate that nanodiamond addition had only a limited effect on the profile roughness of the investigated surfaces. The discrepancy between the Sa and Ra results may be attributed to the different nature of the evaluated parameters. While Sa reflects the three-dimensional surface topography over the entire measured area, Ra is derived from a two-dimensional profile and may therefore be less sensitive to subtle changes in surface morphology caused by nanodiamond incorporation.

### 3.4. Coefficient of Friction (CoF)

**Figure 4:**
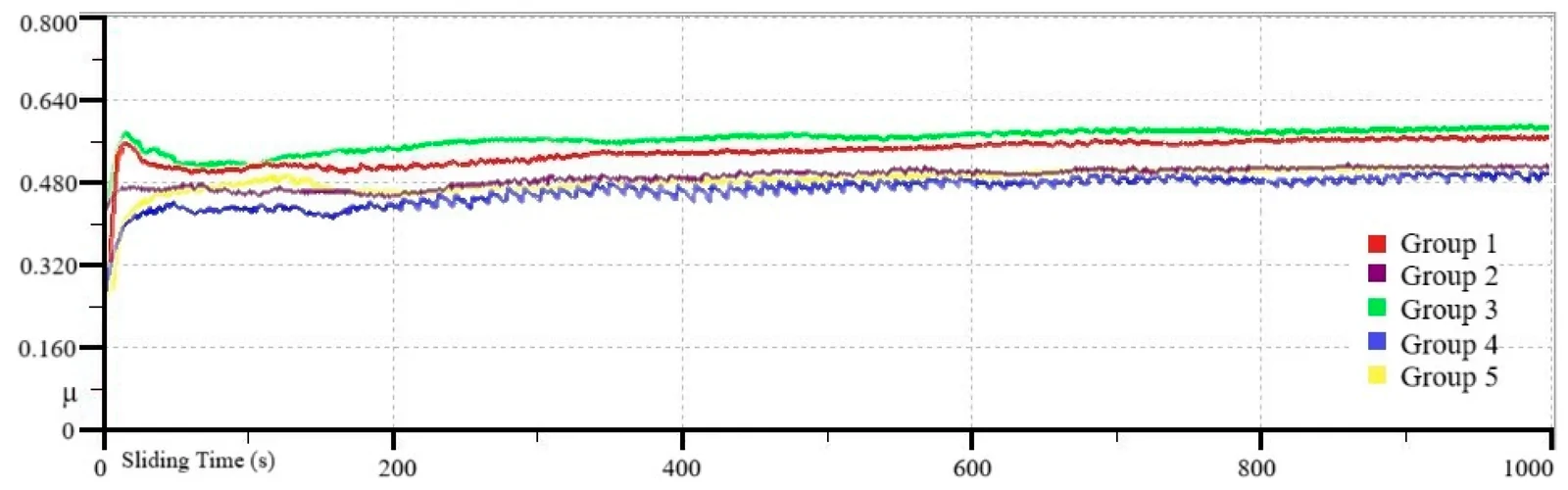
The evolution of the friction coefficient of samples over time.

The average steady-state coefficient of friction (CoF) varied among the investigated groups, indicating that nanodiamond incorporation influenced the tribological behaviour of the glass ionomer cement. group 1 and group 3 exhibited the highest steady-state CoF (0.552), whereas the lowest CoF was recorded for group 4 (0.444). Following an initial run-in period, the friction curves gradually stabilized and reached a relatively steady state for all formulations (Figure 4).

Among the modified materials, groups 2, 4, and 5 consistently exhibited lower CoF values throughout the test duration compared with the reference material (Table 3). Statistical analysis revealed a significant effect of nanodiamond modification on the coefficient of friction (CoF), as confirmed by one-way ANOVA (*F*(4,10) = 11.11, *p* = 0.01056, *η*^2^ = 0.90), indicating a very large effect size. Subsequent Tukey’s HSD post hoc analysis demonstrated that the CoF values of groups 2, 4, and 5 differed significantly from those of the control (group 1) (*p* < 0.05), whereas no statistically significant difference was observed between group 3 and the reference material (*p* > 0.05). The lowest coefficient of friction obtained for group 4 suggests that the incorporation of 2 wt.% nanodiamonds provided the most favourable tribological response among the investigated formulations. This improvement may be associated with changes in surface morphology, more effective load distribution within the cement matrix, or the formation of a more stable contact interface during sliding. In contrast, the friction behaviour of group 3 remained comparable to that of the unmodified material, indicating that the beneficial effect of nanodiamond addition is strongly dependent on its concentration.

### 3.5. Wear Track

**Figure 5:**
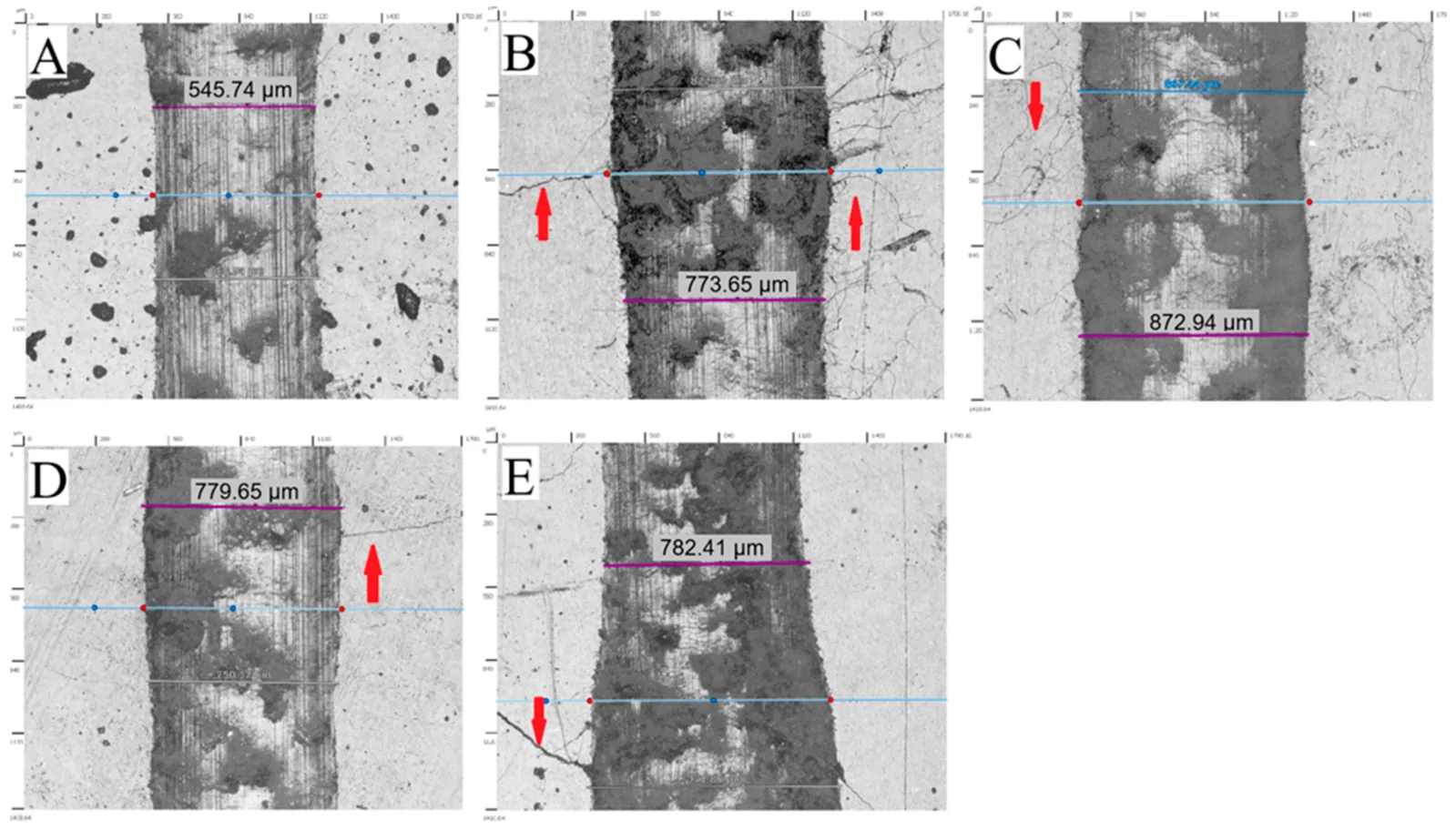
Images of tribological test traces (**A**) GIC without modification; (**B**) GIC + 0.5 wt.%; (**C**) GIC + 1 wt.%; (**D**) GIC + 2 wt.%; (**E**) GIC + 4 wt.%. The red arrows indicate locations in the modified groups where the reduction in microhardness likely contributed to material fractures during sliding contact. The red/blue dot and lines of various colours are included solely to illustrate the width of the tribological wear mark directly in the figure. They are not intended to represent additional data or different experimental conditions.

Wear track analysis revealed substantial differences in wear resistance among the investigated groups (Figure 5). Group 5 exhibited the largest wear track width (908.76 µm) and the greatest wear depth (83.47 µm), indicating the highest degree of surface degradation during tribological testing. Similarly, groups 2 and 3 showed relatively pronounced wear, although to a lesser extent than group 5. In contrast, the unmodified GIC (group 1) exhibited the narrowest wear tracks (652.06 µm) and lowest wear depth (29.77 µm), demonstrating the highest wear resistance among all tested materials. Interestingly, despite exhibiting one of the highest CoF, the reference material showed the lowest material loss, suggesting that friction coefficient and wear resistance were not directly correlated in the investigated system. One-way ANOVA revealed significant differences among the groups for wear track width (*F*(4,10) = 14.14, *p* = 0.003273, *η*^2^ = 0.90) and wear depth (*F*(4,10) = 6.995, *p* = 0.01911, *η*^2^ = 0.82), indicating very large effect sizes for both parameters. Subsequent Tukey’s HSD post hoc analysis demonstrated that all nanodiamond modified groups differed significantly from the control group 1 (*p* < 0.05), both in terms of wear track width and wear depth. The progressive increase in wear observed in the modified formulations suggests that nanodiamond addition did not enhance the wear resistance of the glass ionomer cement under the applied testing conditions. This behaviour may be associated with changes in the integrity of the cement matrix, reduced microhardness, or insufficient bonding between the nanodiamond particles and the surrounding matrix, which could facilitate material removal during sliding contact.

## 4. Discussion

**Figure 6:**
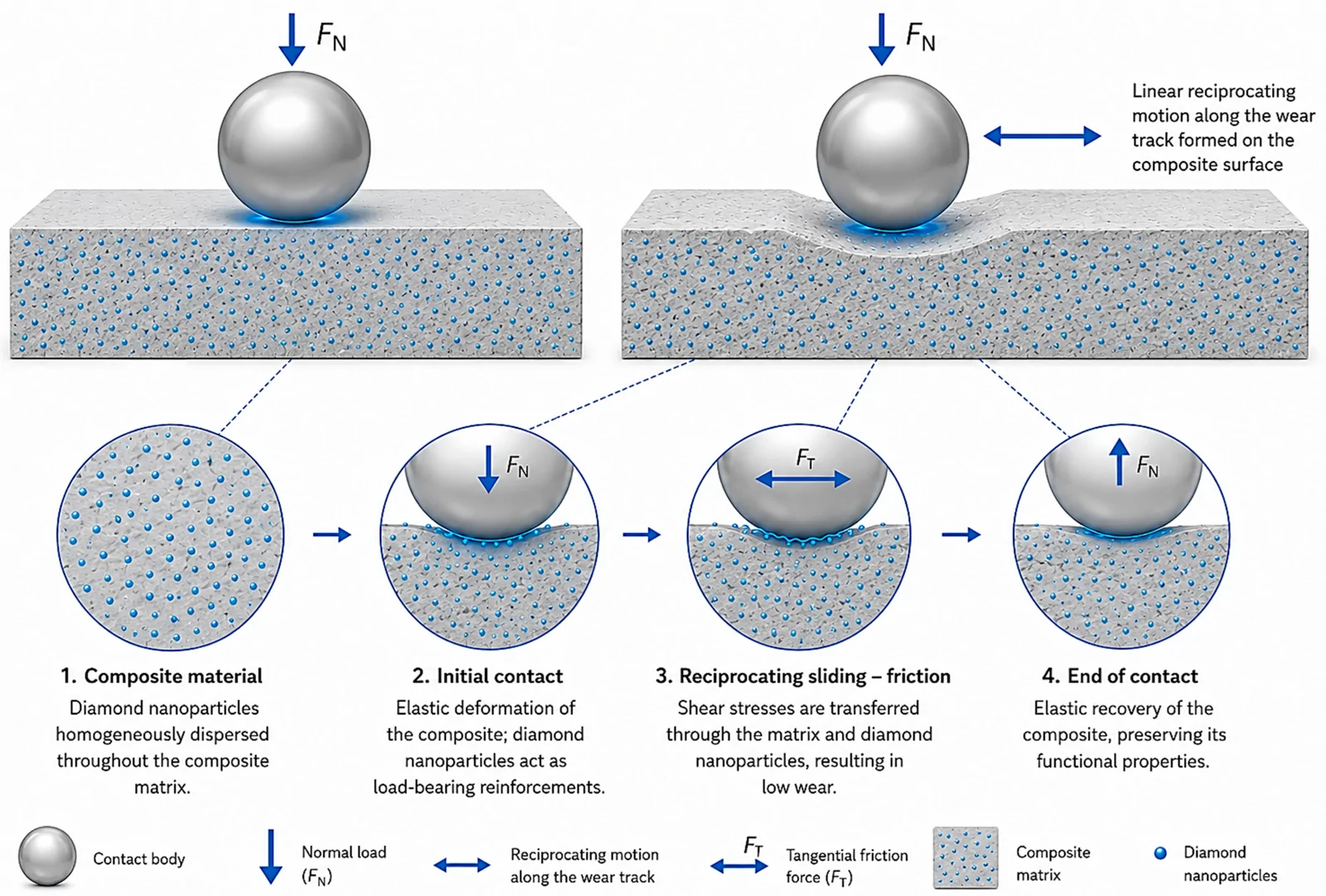
A schematic diagram of the proposed mechanistic model illustrating the hypothesized interaction between the nanodiamond-modified GIC matrix and a moving mating component, potentially resulting in a reduction in the coefficient of friction.

The incorporation of nanodiamonds (NDs) into glass ionomer cement (GIC) significantly influenced both the mechanical and tribological behavior of the material. The addition of NDs, particularly at concentrations of 0.5, 2, and 4 wt.%, resulted in a noticeable reduction in the coefficient of friction. This effect may be attributed to the unique surface properties of nanodiamonds, which can reduce interfacial shear stresses and facilitate smoother sliding at the contact interface (Figure 6). The measured coefficients of friction and the overall tribological behavior were in good agreement with values reported in previous studies investigating nanoparticle-modified dental materials and glass ionomer cements [35,36,37].

Despite the beneficial reduction in friction, the incorporation of nanodiamonds did not lead to an improvement in the mechanical performance of the material. On the contrary, a significant decrease in Vickers microhardness was observed following nanoparticle addition. The most pronounced reduction occurred in groups 3 and 5, corresponding to ND concentrations of 1.0 and 4.0 wt.%, respectively. Similar microhardness values for conventional GICs have been reported in other studies [38,39,40,41]. Furthermore, several studies have demonstrated that the incorporation of nanoparticles above an optimal concentration may adversely affect the microstructure of the cement matrix and consequently reduce surface hardness [42,43,44,45,46,47]. We hypothesize that the reduction in microhardness and wear resistance can also be explained by the potential interference of nanodiamonds with the classic acid-base setting reaction of the glass ionomer cement. Water plays an absolutely critical role in the GIC setting process; it serves as the reaction medium that allows the ionisation of polyalkenoic acid and the transport of leached metal ions (Al^3+^, Sr^2+^) to form the cross-linked polycarboxylate matrix [46]. Because detonation nanodiamonds possess an immense specific surface area decorated with hydrophilic oxygen-containing functional groups (such as hydroxyl and carboxyl groups) [47], they strongly absorb and compete for the available water within the cement mixture. This competition can prematurely dehydrate the local reaction zones, leading to incomplete ionization of the polyacid and a lower degree of cross-linking. Furthermore, the presence of nanodiamond agglomerates creates significant steric hindrance. These clusters physically obstruct the polymer chains from efficiently wrapping around and bonding with the fluoro-aluminosilicate glass particles. The combination of local water depletion and steric blocking results in a less cohesive, structurally compromised polycarboxylate network, which directly manifests as reduced surface microhardness and increased susceptibility to wear.

Because the nanodiamonds were added to the original GIC powder rather than substituted for a fraction of it, the overall powder-to-liquid ratio was inevitably altered. This additive approach decreased the relative volume of liquid available for the reaction, further exacerbating the incomplete ionization of the polyacid and contributing to the reduced mechanical strength.

One of the possible explanation for the observed decrease in microhardness is the tendency of nanodiamond particles to agglomerate during powder mixing. The observed reduction in microhardness, particularly at higher nanodiamond concentrations (2–4 wt.%), is closely linked to the inherent physical properties of detonation nanodiamonds (DNDs). From a materials science perspective, unmodified DNDs possess an exceptionally high specific surface area and high surface energy, along with abundant oxygen-containing surface functional groups. This creates a strong thermodynamic driving force for the nanoparticles to minimise their surface energy by forming tight agglomerates via van der Waals forces and inter-particle hydrogen bonding [47,48]. Consequently, achieving a true nanoscale dispersion of pristine DNDs in a solid powder matrix without prior surface functionalization or the use of specific dispersing agents is highly challenging. At concentrations exceeding 1–2 wt.%, the probability of nanoparticle clustering increases significantly. It is suggested that these agglomerates act as structural defects and stress concentrators within the polyalkenoate matrix, potentially disrupting the acid-base setting reaction and weakening the cohesive strength of the cement, which may explain the recorded drop in both microhardness and wear resistance.

The formation of agglomerates may hinder homogeneous particle distribution, generate local structural defects, and reduce the effectiveness of stress transfer within the matrix. In addition, excessive nanoparticle content may interfere with the acid–base setting reaction of the glass ionomer cement, resulting in a less compact and mechanically weaker structure. These mechanisms may explain why higher nanodiamond concentrations did not produce the expected reinforcing effect.

**Figure 7:**
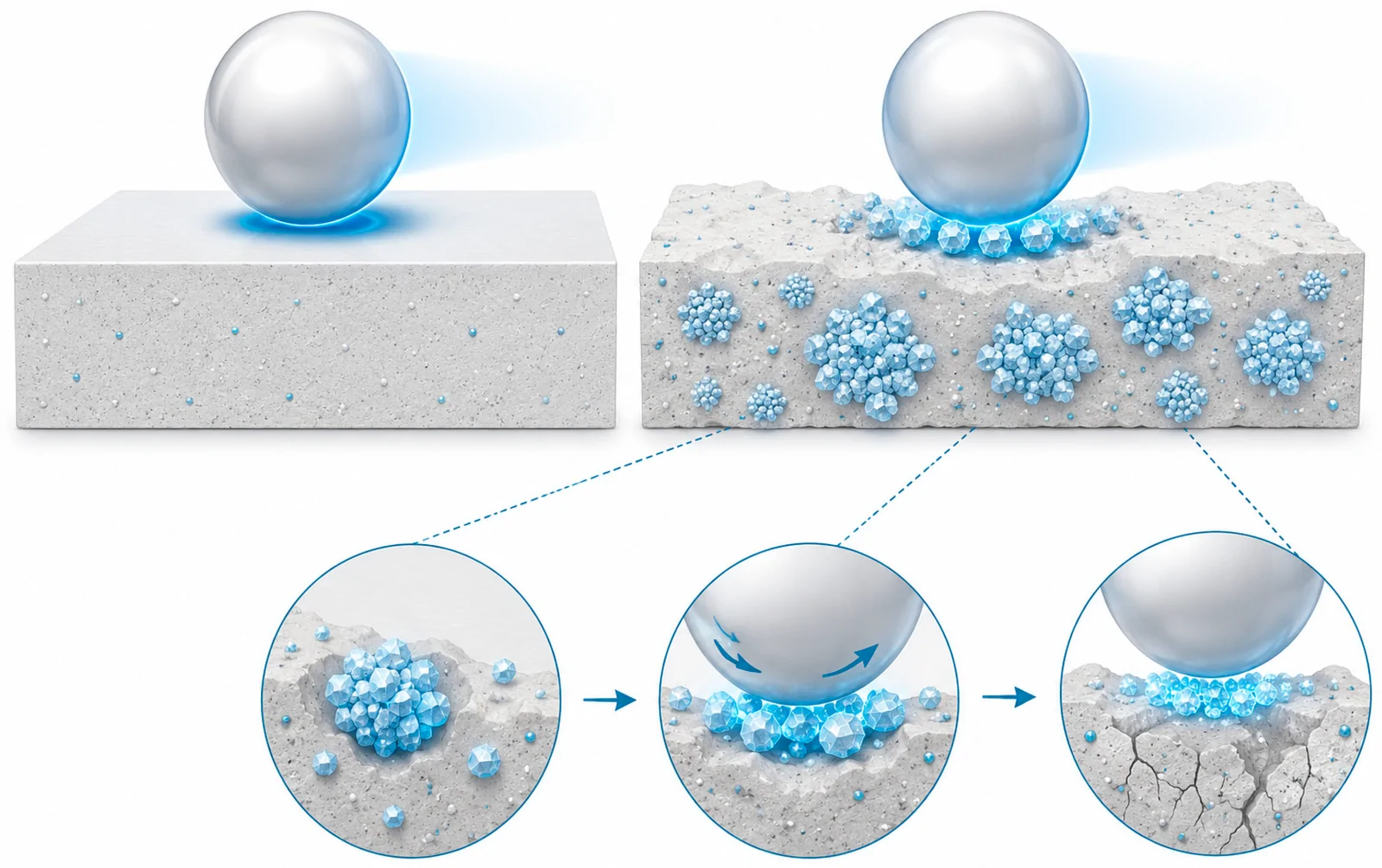
Schematic representation of the proposed mechanistic model illustrating the hypothesized interaction between the nanodiamond-modified GIC matrix and the sliding counterbody, highlighting the potential third-body wear mechanism.

We hypothesize that the relationship between the reduced microhardness and the fluctuating trend observed in the coefficient of friction curves may be related to a third-body wear mechanism. If the incorporation of nanodiamonds disrupted the setting reaction and yielded a softer polycarboxylate matrix, the surface would become more susceptible to initial mechanical degradation during sliding contact. Consequently, we suggest that dislodged fragments of the softened cement matrix, along with potentially released nanodiamond agglomerates, became trapped at the sliding interface. These trapped particles likely acted as abrasive third bodies. The intermittent trapping, rolling, and crushing of this micro-debris between the counterbody and the sample surface could cause the localized spikes and continuous fluctuations in the friction curves. This proposed mechanistic model of interaction—highlighting the physical trade-off where nanodiamonds may reduce overall friction but simultaneously induce third-body wear due to potential matrix softening—is visually summarized in the schematic diagram (Figure 7).

The tribological results further revealed that the reduction in the coefficient of friction was not directly associated with improved wear resistance. Although several modified groups exhibited lower friction coefficients, all nanodiamond-containing formulations showed significantly greater wear than the unmodified cement. This finding suggests that wear resistance in GICs is governed not only by frictional behavior but also by the integrity, hardness, and cohesion of the cement matrix. Consequently, the reduced microhardness observed in the modified groups likely contributed to the increased material removal during sliding contact.

The morphology of the wear tracks (Table 3 and Figure 5A–E) provided further insight into the wear mechanisms operating in the investigated materials. The modified glass ionomer cements generally exhibited wider and deeper wear scars than the unmodified reference material, which is consistent with the quantitative wear measurements. The reference cement (Figure 5A) displayed a relatively narrow and uniform wear track with a smooth surface morphology and only minor signs of local damage, indicating good resistance to material removal during sliding. In contrast, the nanodiamond-modified samples, particularly groups 2 and 5 exhibited more pronounced surface deterioration, characterized by increased material loss, microcrack formation, local fracture events, and partial delamination along the edges of the wear track. The presence of these defects suggests that the incorporation of nanodiamonds may have altered the structural integrity of the cement matrix. One possible explanation is the formation of nanoparticle agglomerates during mixing, which can act as stress concentrators and promote crack initiation under repeated mechanical loading. Furthermore, weak interfacial bonding between the nanodiamond particles and the surrounding matrix may facilitate particle pull-out and local material fragmentation during sliding contact. These observations are in agreement with the microhardness and wear results, which indicated reduced resistance to mechanical degradation in the modified groups. Consequently, the wear-track morphology supports the hypothesis that, although nanodiamond addition contributed to a reduction in the coefficient of friction, it simultaneously decreased the structural cohesion of the cement matrix, resulting in accelerated wear. This finding highlights the complex relationship between friction reduction and wear resistance in nanoparticle-reinforced glass ionomer cements.

Surface roughness analysis provided additional insight into the influence of nanodiamond incorporation on the surface characteristics of glass ionomer cements. The areal roughness parameter Sa exhibited statistically significant differences among the investigated groups (*p* < 0.05). Post hoc analysis revealed that only group 5 differed significantly from the reference material (*p* = 0.00058), whereas the remaining groups showed no statistically significant differences compared with the control cement. The highest Sa values were recorded for group 1 (383.7 ± 154.3 nm) and group 4 (380.8 ± 142.1 nm), while the lowest value was observed in group 5 (147.5 ± 42.5 nm). This substantial reduction in surface roughness at the highest nanodiamond concentration suggests the formation of a more compact and uniform surface morphology. A smoother surface may reduce the degree of mechanical interlocking between contacting surfaces and consequently contribute to the observed reduction in the coefficient of friction. Therefore, the lower friction coefficients measured in some nanodiamond-modified groups may be partially attributed to changes in surface topography in addition to the intrinsic properties of the nanoparticles. In contrast, the profile roughness parameter Ra did not reveal any statistically significant differences among the investigated groups (*p* > 0.05). Nevertheless, minor variations in the mean values were observed, with the highest Ra recorded for group 3 (25.8 ± 4.7 nm) and the lowest for group 2 (22.5 ± 7.5 nm). The absence of significant differences in Ra, despite the significant changes detected for Sa, highlights the different sensitivity of areal and profile roughness parameters to subtle modifications of surface morphology. When compared with previously published studies, the Ra values obtained in the present work were approximately one order of magnitude lower than those commonly reported for conventional glass ionomer cements [49,50,51,52]. This discrepancy is likely related to differences in measurement methodology. Most previous studies employed contact profilometry, which evaluates roughness along a single line and may be influenced by stylus geometry and measurement conditions. In contrast, the present study used confocal microscopy, a non-contact optical technique capable of capturing three-dimensional surface topography with high spatial resolution. Similar Ra values have been reported in studies employing optical or confocal surface characterization methods, supporting the reliability of the results obtained in the present investigation [39]. Taken together, the roughness results indicate that nanodiamond incorporation primarily affected the three-dimensional surface morphology of the cement rather than the profile roughness itself.

These findings further support the hypothesis that modifications in surface topography contributed to the observed changes in tribological behavior. The EDX analysis revealed a slight increase in carbon content in the nanodiamond-modified samples, which is consistent with the incorporation of carbon-based nanoparticles into the glass ionomer cement matrix. However, it should be noted that EDX measurements performed in a scanning electron microscope may also be influenced by surface carbon contamination, a commonly reported artefact associated with sample handling, environmental exposure, or hydrocarbon deposition during analysis. Therefore, the measured increase in carbon content should be interpreted with caution. In contrast, the concentrations of the principal elements associated with the glass phase of the cement, namely (F, Al, Si) remained relatively unchanged across all experimental groups. The observed elemental composition was in good agreement with values previously reported for conventional glass ionomer cements and nanoparticle-modified formulations [53,54,55,56]. The stability of the major elemental constituents indicates that nanodiamond incorporation did not substantially alter the chemical composition of the glass ionomer matrix. Consequently, the changes observed in microhardness, surface roughness, friction behavior, and wear resistance are unlikely to be associated with chemical modifications of the cement network. Instead, these effects are more likely governed by physical factors, including nanoparticle dispersion, agglomeration phenomena, interfacial interactions between nanodiamonds and the cement matrix, and their influence on microstructural development during the setting process. This interpretation is further supported by the absence of significant changes in the concentrations of the key glass-forming elements, suggesting that the fundamental acid–base reaction responsible for cement formation remained largely unaffected by nanodiamond addition.

These observations are consistent with the growing body of recent literature, which highlights the complex structural and mechanical trade-offs encountered when incorporating nanodiamonds and other carbon-based nanomaterials into GIC matrices [55,56,57,58].

Based on these results, the existence of an optimal ND concentration can be hypothesized, most likely within the range of 0.5–2 wt.%, where improved tribological performance may be achieved while limiting the adverse effects on the mechanical properties of the glass ionomer cement. Among the investigated formulations, the sample containing 2 wt.% ND exhibited the lowest coefficient of friction, suggesting that this concentration may provide the most favorable balance between friction reduction and material performance.

The results further indicate that the effectiveness of nanodiamond reinforcement is governed not only by nanoparticle concentration but also by their dispersion quality, distribution within the matrix, and interfacial interaction with the surrounding cement structure. Insufficient dispersion may promote nanoparticle agglomeration, leading to local stress concentrations, structural heterogeneities, and premature material degradation under mechanical loading.

Future research should therefore focus on improving the compatibility between nanodiamonds and the glass ionomer matrix. Particular attention should be devoted to surface functionalization of nanodiamond particles, which may enhance their chemical affinity to the matrix, improve particle dispersion, and reduce the tendency for agglomerate formation. In addition, advanced dispersion techniques, such as ultrasonic treatment or high-energy mixing methods, should be investigated to achieve a more homogeneous nanoparticle distribution throughout the material.

Overall, whilst nanodiamonds represent a fascinating nanofiller capable of reducing the coefficient of friction of glass ionomer cements (GICs), their tendency to agglomerate and reduce overall microhardness poses a significant obstacle to immediate clinical application. Before their clinical suitability can be established, future formulations must be rigorously tested in accordance with the ISO 9917-1:2025(en) [59] standard for acid-base cements. However, successful implementation requires careful optimization of nanoparticle concentration, surface chemistry, and dispersion strategy to ensure that improvements in frictional performance are not accompanied by deterioration of mechanical integrity. Such optimization will be essential for the future clinical application of nanodiamond-reinforced glass ionomer restorative materials.

## 5. Limitations and Future Directions

While this study provides valuable insights into the macroscopic tribological and mechanical properties of ND-modified GICs, it is not without limitations. Neither the precise chemical kinetics of the curing reaction nor the degree of cross-linking of the material have been directly determined.

Future studies should focus on advanced physicochemical characterisation, including Fourier-transform infrared spectroscopy (FTIR) and nuclear magnetic resonance (NMR), to deeply monitor the setting reaction kinetics. Additionally, surface functionalization strategies for nanodiamonds should be investigated to improve their chemical affinity to the polyalkenoate matrix and mitigate agglomeration, aiming to achieve friction reduction without compromising the structural integrity of the cement.

Furthermore, the clinical relevance of these preliminary findings must be interpreted with caution. The current study is limited to short-term in vitro tribological testing. Before any clinical applicability can be considered, future research must comprehensively evaluate compressive and flexural strength, shear bond strength to dentin and enamel, long-term fluoride release, water sorption, and biocompatibility. Additionally, simulating the oral environment through thermocycling, pH cycling, and testing in artificial saliva or aging media is strictly required.

## 6. Conclusions

This study evaluated the influence of nanodiamond (ND) addition on the mechanical, surface, and tribological properties of glass ionomer cement (GIC). The results showed that ND incorporation reduced the coefficient of friction and modified the surface morphology, particularly at higher concentrations. However, these improvements were accompanied by a decrease in microhardness and wear resistance compared with the unmodified cement.

Preliminary EDX analysis suggested that the principal chemical composition of the GIC matrix remained largely unchanged, which supports the hypothesis that the observed effects were primarily related to physical interactions between the nanodiamonds and the cement matrix.

Overall, nanodiamonds show potential as functional additives for tribological modification of GICs, although further optimization of nanoparticle dispersion and surface functionalization is required to improve their structural integrity effect and clinical applicability.

## Figures and Tables

**Table 1:** Elemental composition determined by EDX analysis in glass-ionomer cements modified with various concentrations of nanodiamonds.

Element (wt.%)	Group 1	Group 2	Group 3	Group 4	Group 5
C	19.42	16.68	17.67	17.26	20.63
O	35.12	35.37	35.00	35.89	33.90
F	7.30	8.42	8.18	8.84	7.24
Na	0.88	0.99	1.11	1.02	1.08
Al	9.81	10.58	10.49	10.44	9.91
Si	10.25	10.78	11.03	10.71	10.54
P	1.46	1.45	1.51	1.46	1.54
S	0.72	0.44	0.32	0.19	0.42
Cl	0.02	0.01	0.03	0.02	0.27
K	0.05	0.00	0.03	0.00	0.03
Ca	0.11	0.12	0.14	0.04	0.05
Cr	0.03	0.05	0.05	0.03	0.06
Fe	0.14	0.14	0.16	0.13	0.19
Sr	14.55	14.70	14.07	13.78	13.92
Ba	0.16	0.23	0.21	0.19	0.21
Total	100	100	100	100	100

**Table 2:** Mean and standard deviation (SD) of the microhardness.

HV 0.1	GR1	GR2	GR3	GR4	GR5
Mean	64.91	46.83	32.29	41.09	31.58
SD	3.10	7.51	5.42	1.82	13.97

**Table 3:** Mean and standard deviation (SD) of the coefficient of friction, width and depth of wear of samples.

	CoF (-) ± SD	Profile Width (µm) ± SD	Profile Depth (µm) ± SD
Group 1	0.552 ± 0.014	652.06 ± 17.85	29.77 ± 2.17
Group 2	0.552 ± 0.018	863.05 ± 10.94	57.6 ± 2.63
Group 3	0.444 ± 0.029	838.37 ± 100.52	55.46 ± 17.15
Group 4	0.483 ± 0.006	908.76 ± 4.85	83.47 ± 22.39
Group 5	0.481 ± 0.031	600.68 ± 33.67	36.04 ± 13.36

## Data Availability

The data are available from the author for correspondence upon reasonable request.

## Abbreviations

The following abbreviations are used in this manuscript:

GICs	Glass ionomer cements
ND	Nanodiamond
EDX	Energy dispersive X-ray spectroscopy
SEM	Scanning electron microscope
ISO	International Standard Organization
HV	Hardness Vickers
ASTM	American Society for Testing and Materials
ASTM G99	Standard Test Method for Wear Testing with a Pin-on-Disk Apparatus
ASTMG133	Standard Test Method for Linearly Reciprocating Ball-on-Flat Sliding Wear
Al2O3	Aluminium oxide
FTIR	Fourier-transform infrared spectroscopy
NMR	Nuclear magnetic resonance
CoF	Coefficient of friction
Sa	Areal roughness parameter
Ra	Profile roughness parameter
